# Complete Molar Pregnancy in a 50-Year-Old Postmenopausal Woman

**DOI:** 10.7759/cureus.80315

**Published:** 2025-03-09

**Authors:** Burhan Vural, Sumeyya Duran Kaymak, Berna Turhan, Rasime Pelin Kavak, Dilek Yüksel

**Affiliations:** 1 Radiology, Etlik City Hospital, Ankara, TUR; 2 Obstetrics and Gynecology, Etlik City Hospital, Ankara, TUR

**Keywords:** abnormal vaginal bleeding, gestational trophoblastic disease (gtd), hydatiform mole, molar pregnancy, postmenopausal period

## Abstract

Gestational trophoblastic diseases (GTDs) are usually seen in women of reproductive age, but rarely, they can also be observed in postmenopausal women. In a postmenopausal woman presenting with abnormal vaginal bleeding, the differential diagnosis of gestational trophoblastic disease using histopathological, clinical, and radiological methods is important to prevent delays in treatment.

A 50-year-old female patient presented to our clinic with complaints of abnormal vaginal bleeding. Ultrasound imaging revealed a mass lesion in the endometrial cavity consistent with a molar pregnancy. Furthermore, the biochemical value of beta-human chorionic gonadotropin (B-HCG) was 353,761 IU/L, supporting the diagnosis. For further evaluation, the patient underwent computed tomography and magnetic resonance imaging. Additional imaging supported the diagnosis. Based on these results, the patient underwent a total abdominal hysterectomy with bilateral salpingo-oophorectomy. Pathology results confirmed a complete molar pregnancy.

In this case report, we present the clinical and radiological approach in the differential diagnosis of gestational trophoblastic disease in a 50-year-old female patient.

## Introduction

Gestational trophoblastic neoplasia is a broad-spectrum disease that includes both benign and malignant processes resulting from the abnormal proliferation of trophoblastic cells. Gestational trophoblastic neoplasia is histopathologically divided into five main groups. These include hydatidiform mole (complete and partial), invasive mole, choriocarcinoma, placental site trophoblastic tumor (PSTT), and epithelioid trophoblastic tumor (ETT) [1]. Hydatidiform mole is an abnormal genetic fertilization and gametogenesis disorder usually seen in women of reproductive age (13-49 years). Molar pregnancy is divided into two types based on its morphological and cytogenetic characteristics: complete and partial [2]. Although different definitions have been made for the incidence of molar pregnancy in various regions, the main rates are approximately 12/1,000 pregnancies (Indonesia, India, and Turkey), 1-2/1,000 pregnancies (Japan and China), and 0.5-1/1,000 pregnancies (North America and Europe) [3]. Maternal age and previous reproductive history are important risk factors for the development of molar pregnancy. Women over the age of 40 have a 5-10 times increased risk for complete molar pregnancy, while nearly one-third of women over the age of 50 experience the development of molar pregnancy [4]. Complete hydatidiform mole is extremely rare in postmenopausal women. Since it was first described, approximately 15 cases have been reported worldwide [5]. This case report aims to highlight the radiological and clinical attention in the diagnosis of molar pregnancy in a 50-year-old postmenopausal woman presenting with abnormal vaginal bleeding.

## Case presentation

A 50-year-old female patient (gravida 16, para 5, living 5, dilation/curettage 10, ectopic pregnancy 1) presented to the gynecology and obstetrics department of our hospital with a complaint of vaginal bleeding that had been ongoing for two days. The patient had coronary artery disease and an umbilical hernia. The patient had no comorbidities, was in good general condition, and had no other additional complaints. Her last menstrual period was one year ago. Physical examination of the vulva and vagina was normal. The uterus and cervix were consistent with multiparity. In biochemistry and complete blood count parameters, a hemoglobin level of 10.2 mg/dL (reference range: 11.5-15 mg/dL) was the only pathological finding. There was no beta-human chorionic gonadotropin (B-HCG) test at the time of the patient's initial visit.

**Figure 1:**
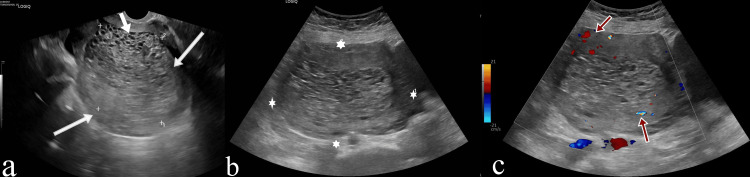
Grayscale transabdominal and transvaginal ultrasound images of the mass lesion (a) In the grayscale transvaginal ultrasound image, a mass lesion with a characteristic vesicular pattern is seen filling almost the entire endometrial cavity (white arrows). (b) In the grayscale transabdominal axial ultrasound image, an endometrial mass measuring approximately 13×9 cm is seen (asterisk). (c) In the axial plane color Doppler ultrasound image, the internal vascularity of the lesion is seen (red arrows).

Transabdominal and transvaginal ultrasound examinations were conducted on the patient. During the ultrasound examination, a heterogeneous mass lesion measuring approximately 13×9 cm, with a spongy component being the dominant feature and also containing a solid component measuring 9×3 cm, was observed filling the endometrial cavity almost completely. Additionally, in the left ovary, a cystic appearance consistent with an endometrioma measuring 2×1 cm with homogeneous dense content was observed. Both adnexal regions and the right ovary were normal (Figure 1).

**Figure 2:**
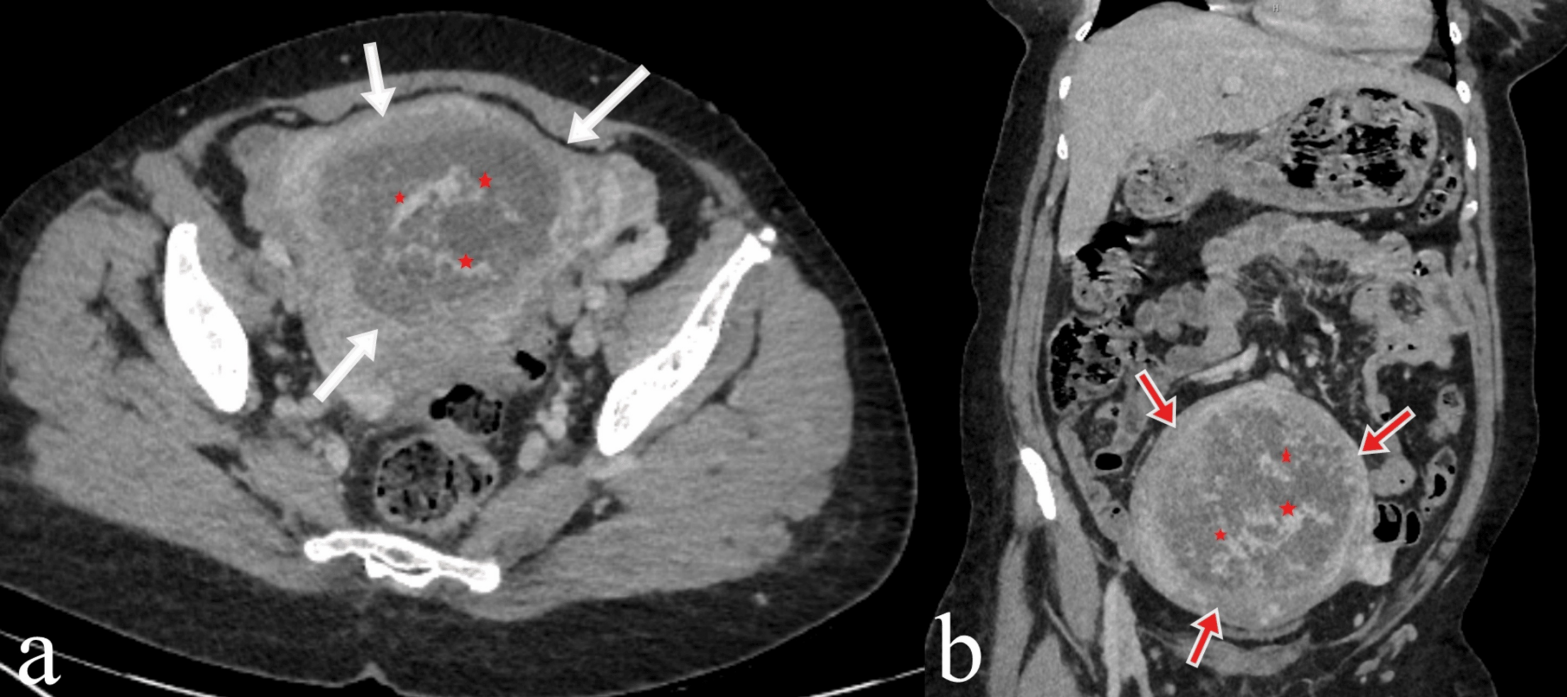
Axial and coronal contrast-enhanced abdominopelvic computed tomography images of the mass lesion (a) Axial and (b) coronal contrast-enhanced abdominopelvic computed tomography images show a heterogeneous mass lesion in the uterus (white and red arrows). There is a more pronounced contrast enhancement in the center of the mass (red asterisk).

The patient was initially evaluated with ultrasound images. Correlation with laboratory values was recommended. Biochemical values showed a B-HCG level of 353.761 IU/L; the diagnosis of gestational trophoblastic disease was considered. Additional imaging, including pelvic-brain magnetic resonance imaging and abdominopelvic-thoracic computed tomography, was used to assess myometrial invasion and exclude metastatic disease. In the performed abdominopelvic computed tomography (Figure 2), thoracic computed tomography, and brain magnetic resonance imaging, no metastatic involvement was observed in the lungs, liver, or brain.

**Figure 3:**
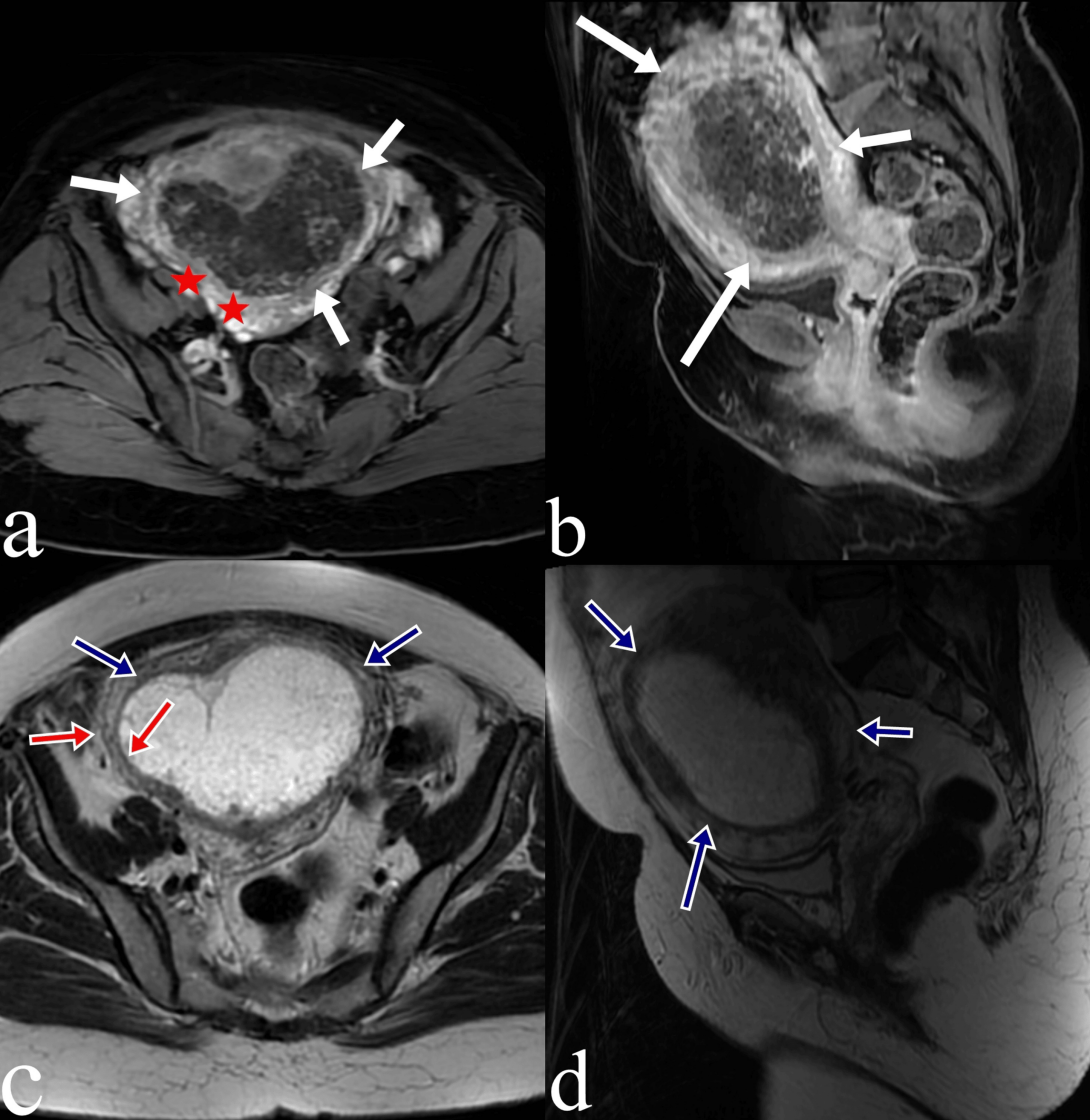
T1- and T2-weighted magnetic resonance images of the mass lesion (a) Axial and (b) sagittal T1-weighted magnetic resonance images show a mass lesion with a more vascular pattern compared to the myometrium (white arrows). Thinning and invasion of the myometrium are observed in the right half of the fundus (red asterisk). (c) Axial and (d) sagittal T2-weighted images show the mass lesion with hyperintense signal characteristics compared to the myometrium (blue arrows) and thinning of the myometrium in the right half of the fundus (red arrows).

In the pelvic magnetic resonance imaging performed, a heterogeneous mass measuring approximately 13×9 cm with a more vascular pattern compared to the myometrium was observed in the endometrial cavity. It was also seen that the mass was mostly confined to the uterus but showed myometrial invasion in some areas, extending to the serosa in the right half of the fundus (Figure 3).

**Figure 4:**
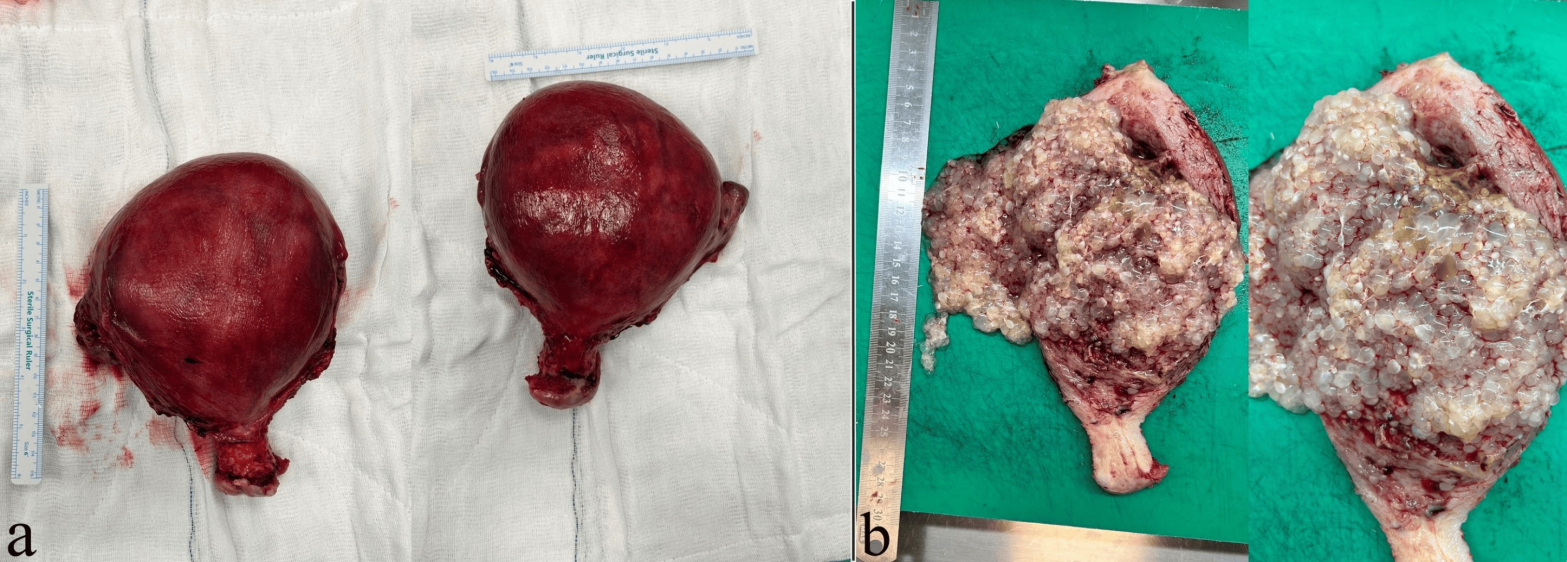
Macroscopic view of the molar tissue (a) The total abdominal hysterectomy and bilateral salpingo-oophorectomy material is observed. The uterus is enlarged. (b) Macroscopic view of the molar tissue. A large amount of grapelike spongy material is seen, containing no fetal parts.

Based on these results, the patient underwent a total abdominal hysterectomy with bilateral salpingo-oophorectomy. The specimen was sent to the pathology department. The pathology specimen consisted of a total abdominal hysterectomy and bilateral salpingo-oophorectomy material measuring 17×12×9 cm. Macroscopic examination revealed a lesion approximately 17×12 cm in size, composed of variably sized vesicular and transparent structures (resembling a bunch of grapes) nearly filling the entire endometrial cavity (Figure 4). Microscopic examination showed generalized hydropic villi and proliferation of trophoblastic cells. Histomorphologically, it was consistent with a complete hydatidiform mole.

**Figure 5:**
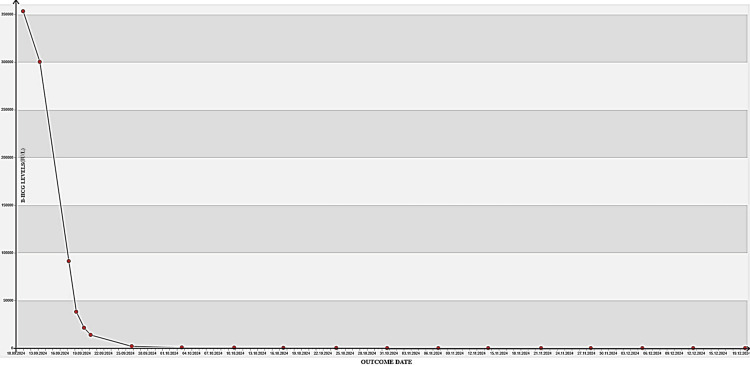
B-HCG levels The regression of the patient's B-HCG levels is observed. B-HCG: beta-human chorionic gonadotropin

During the patient's postoperative follow-up, B-HCG levels were observed to regress significantly: 91,417 IU/L on postoperative day 1, 2,257 IU/L in week 1, 2,580 IU/L in week 3, and 0 IU/L in week 4 (Figure 5).

## Discussion

Although gestational trophoblastic neoplasia is seen in women of reproductive age (13-49 years), it may be seen rarely in postmenopausal women. Literature reviews of the few cases reported show that the majority of patients primarily presented with vaginal bleeding (80%). Less frequently, abdominal pain, nausea, and vomiting may also occur [6,7].

Ultrasonography plays an important role in the evaluation of a complete mole. Chorionic villi and diffuse hydropic swelling are characteristically seen on ultrasound as numerous echoes with a vesicular pattern and holes within the placenta [7,8].

B-HCG is a specific marker for gestational trophoblastic diseases. In the hydatidiform spectrum, high B-HCG values are often observed. In more than half of the patients, pre-treatment B-HCG levels are above 100,000 IU/L [9].

Computed tomography, although limited in assessing the primary disease, plays an important role in evaluating and staging metastatic disease. Magnetic resonance imaging is useful in evaluating extrauterine mass extension and pelvic lymph nodes that are not clearly assessed by ultrasonography. Additionally, in evaluating the primary mass compared to normal myometrium, iso-intense signals on T1-weighted images and hyperintense signals on T2-weighted images can contribute to the diagnosis [10].

The main modalities in the treatment of the disease include curettage, suction curettage, chemotherapy, and hysterectomy. In high-risk groups, particularly in women over the age of 50, hysterectomy is generally recommended to prevent malignant processes [11-13].

In conclusion, although a complete hydatidiform mole is rare in patients presenting with postmenopausal vaginal bleeding, it should be considered in the differential diagnosis. Ultrasonographic examination, cystic endometrial changes, and serum B-HCG values significantly contribute to the diagnosis. Computed tomography and magnetic resonance imaging are helpful in the diagnostic process and in evaluating additional pathologies.

## Conclusions

Gestational trophoblastic diseases (GTDs) are usually seen in women of reproductive age, but rarely, they can also be observed in postmenopausal women. In a postmenopausal woman presenting with abnormal vaginal bleeding, the differential diagnosis of gestational trophoblastic disease using histopathological, clinical, and radiological methods is important to prevent delays in treatment.
